# Evaluation of case management of uncomplicated malaria in Haiti: a national health facility survey, 2012

**DOI:** 10.1186/s12936-015-0901-2

**Published:** 2015-10-09

**Authors:** Keren Z. Landman, Samuel E. Jean, Alexandre Existe, Eniko E. Akom, Michelle A. Chang, Jean Frantz Lemoine, Kimberly E. Mace

**Affiliations:** Malaria Branch, Division of Parasitic Diseases and Malaria, Center for Global Health, US Centers for Disease Control and Prevention, Atlanta, GA USA; Population Services International/Organisation Haïtienne de Marketing Social pour la Santé, Port-au-Prince, Haiti; Laboratoire National de Santé Publique/Ministère de la Santé Publique et de la Population (MSPP), Port-au-Prince, Haiti; Programme National de Contrôle de la Malaria/MSPP, Port-au-Prince, Haiti

**Keywords:** Malaria, *Plasmodium falciparum*, Haiti, Case management, Diagnostic tests, Routine, Guideline adherence

## Abstract

**Background:**

Malaria is a public health concern in Haiti, although there are limited data on its burden and case management. National malaria guidelines updated in 2012 recommend treatment with chloroquine and primaquine. In December 2012, a nationally-representative cross-sectional survey of health facilities (HFs) was conducted to determine malaria prevalence among febrile outpatients and malaria case management quality at baseline before scale-up of diagnostics and case management training.

**Methods:**

Among all 833 HFs nationwide, 30 were selected randomly, in proportion to total HFs per region, for 2-day evaluations. Survey teams inventoried HF material and human resources. Outpatients of all ages were screened for temperature >37.5 °C or history of fever; those without severe symptoms were consented and enrolled. Providers evaluated and treated enrolled patients according to HF standards; the survey teams documented provider-ordered diagnostic tests and treatment decisions. Facility-based test results [microscopy and malaria rapid diagnostic tests (RDTs)] were collected from HF laboratories. Blood smears for gold-standard microscopy, and dried blood spots for polymerase chain reaction (PCR) were obtained.

**Results:**

Malaria diagnostic capacity, defined as completing a test for an enrolled patient or having adequate resources for RDTs or microscopy, was present in 11 (37 %) HFs. Among 459 outpatients screened, 257 (56 %) were febrile, of which 193 (75 %) were eligible, and 153 (80 %) were enrolled. Among 39 patients with facility-level malaria test results available on the survey day, 11 (28 %) were positive, of whom 6 (55 %) were treated with an anti-malarial. Twenty-seven (95 %) of the 28 patients testing negative were not treated with an anti-malarial. Of 114 patients without test results available, 35 (31 %) were presumptively treated for malaria. Altogether, 42 patients were treated with an anti-malarial, one (2 %) according to Haiti’s 2012 guidelines. Of 140 gold-standard smears, none were positive, although one patient tested positive by PCR, a more sensitive technique. The national prevalence of malaria among febrile outpatients is estimated to be 0.5 % (95 % confidence interval 0–1.7 %).

**Conclusions:**

Malaria is an uncommon cause of fever in Haitian outpatients, and limited, often inaccurate, diagnostic capacity at baseline contributes to over diagnosis. Scale-up of diagnostics and training on new guidelines should improve malaria diagnosis and treatment in Haiti.

## Background

Haiti and the Dominican Republic share the island of Hispaniola, the only Caribbean island where malaria remains endemic. Despite a significant malaria eradication effort in the 1960s, which dramatically reduced its prevalence, malaria remains a public health challenge for Hispaniola. Funding for malaria activities in Haiti has been limited since 1988 when the National Malaria Eradication Service (Service Nationale de l’Eradication de la Malaria) was incorporated into the primary care system. However, in 2011 a five-year award was granted by the Global Fund to Fight AIDS, Tuberculosis and Malaria (GFATM) to improve malaria control in Haiti. This award committed funds to multiple priorities, including: national distribution of long-lasting insecticide-treated mosquito nets; deployment of widespread vector-control measures; improvement of malaria diagnostics; updating the national treatment guidelines; and strengthening malaria case management [1].

Malaria case management, which is comprised of testing patients with suspected malaria and providing appropriate treatment to confirmed case-patients, is a central element of malaria control. In 2010, the Haitian Ministry of Public Health and Population (Ministère de la Santé Publique et de la Population, MSPP) approved three brands of malaria rapid diagnostic tests (RDTs) to extend diagnostic capacity beyond microscopy [2]. Furthermore, in 2012, Haiti revised its malaria diagnosis and treatment guidelines, adding recommendations to confirm all cases of suspected malaria by a parasitological diagnostic test and to include a single dose of primaquine (0.75 mg/kg) in combination with standard chloroquine therapy (25 mg/kg total, administered over 3 days) for treatment of all confirmed malaria cases. Prior to this revision, it was typical to diagnose malaria based on clinical symptoms including history of fever with or without other symptoms such as nausea, vomiting, diarrhoea, headache, back pain, chills, and myalgia, for which other obvious causes were excluded. The recommendation for this two-drug regimen was made with the goal of reducing malaria transmission via the gametocytocidal activity of primaquine [1], and aligned the malaria treatment policies across Hispaniola [3].

**Fig. 1:**
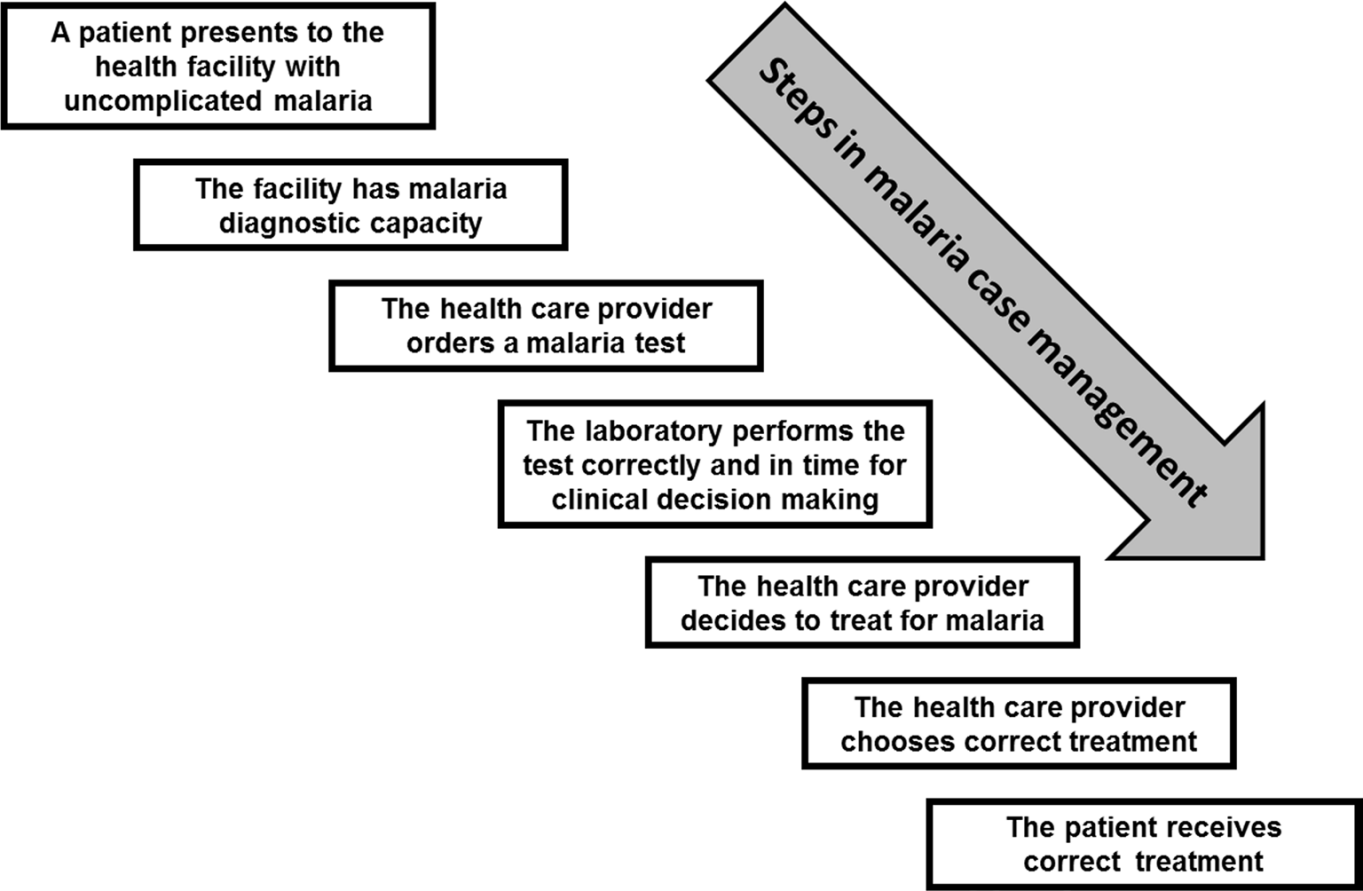
Steps in malaria case management

Multiple health system factors play a role in malaria case management (Fig. 1), and many of these same elements have been historically under-resourced in Haiti. Although high-quality historical data on many health system factors are lacking for Haiti, a few evaluations of diagnostic capacity at local health facilities have demonstrated a low positive predictive value of microscopy at health facility laboratories (22 and 40 %) compared to reference laboratories [4, 5]. Understanding the barriers to good case management should facilitate appropriate resource targeting for programme improvement.

A key indicator to guide malaria control programmes is malaria infection prevalence measured in two populations: (1) febrile patients seeking care at health facilities, measured by routine surveillance or health facility-based surveys; and (2) community members who are generally asymptomatic, measured by household surveys. Previous surveys in health facilities and communities have provided a range of estimates. In Haiti, health facility surveys, conducted in 1995, 2005, and 2007, generally using non-representative sampling designs, reported microscopy-based malaria prevalence rates among febrile outpatients that were between 3.5 and 4.9 % [3–5]. A convenience sample of febrile persons presenting to a non-representative sample of mobile clinics in the months immediately following the 2010 earthquake demonstrated a malaria infection prevalence of 20.3 % by RDT [6]. Community malaria assessments conducted along the coastline of Haiti’s southern peninsula identified parasitemic individuals [7, 8]. A community-based survey conducted in the Artibonite Valley during the rainy season of 2006 estimated a 3.1% prevalence of malaria by polymerase chain reaction (PCR) [9]. In 2011, a national community survey showed that, by all methods (microscopy, RDT and PCR), less than one percent of persons were parasitemic [10].

In late 2012, the MSPP through the national malaria control programme (Programme National de Contrôle de la Malaria, PNCM) and the national public health laboratory (Laboratoire National de Santé Publique, LNSP), in partnership with Population Services International (PSI) and the US Centers for Disease Control and Prevention (CDC) conducted a survey to measure malaria prevalence among febrile patients seeking care at health facilities, and to evaluate diagnostic and treatment practices. This survey was funded by the GFATM malaria grant with the intention of providing baseline values for key programmatic indicators before widespread dissemination of training on the revised guidelines and RDTs.

## Methods

### Survey context and design

According to Haiti’s 2011 health facility census, its health care system includes approximately 800 health facilities distributed among 10 departments [11]. About half of these facilities are *dispensaires* (outpatient clinics typically staffed by those with a nursing degree equivalent), and about one quarter are *centres sans lits* (outpatient clinics often with limited beds for short observation and staffed by at least one physician). The remainder are *centres avec lits* (facilities with inpatient services and one or more physician) or hospitals (facilities with inpatient wards, and specialized services and staff). All types of facilities offer outpatient services. While most hospitals have laboratories on-site, laboratory capacity varies among other types of health facilities.

### Study design

This nationally representative, cross-sectional survey of febrile outpatients used a stratified cluster-sampling design where the primary sampling units, or clusters, were facilities in each department (or stratum). From each stratum, a sample of health facilities was randomly selected in proportion to the number of total facilities in each department. The sampling frame of health facilities for this evaluation included 833 functional facilities with outpatient services, identified in the MSPP national health facility census report of 2011 [11]. All eligible febrile patients presenting for outpatient consultations at the sampled health facility during regular working hours on the survey day were eligible for enrolment. Reliable patient volume data was not available when the survey was planned; therefore, facilities were not selected in proportion to their utilization.

### Enrolment

Criteria for patient enrolment in the study were: (1) experiencing fever, defined as a measured temperature ≥37.5 °C, or history of fever at any time during the previous 2 weeks, (2) being aged 18 years or older, or having a guardian present who was 18 years or older, and (3) providing informed consent. Patients were excluded from participation if they had signs of severe disease, specifically: impaired consciousness, prostration, intractable vomiting, convulsions, respiratory distress, shock, jaundice, or spontaneous bleeding [12].

### Sample size

**Fig. 2:**
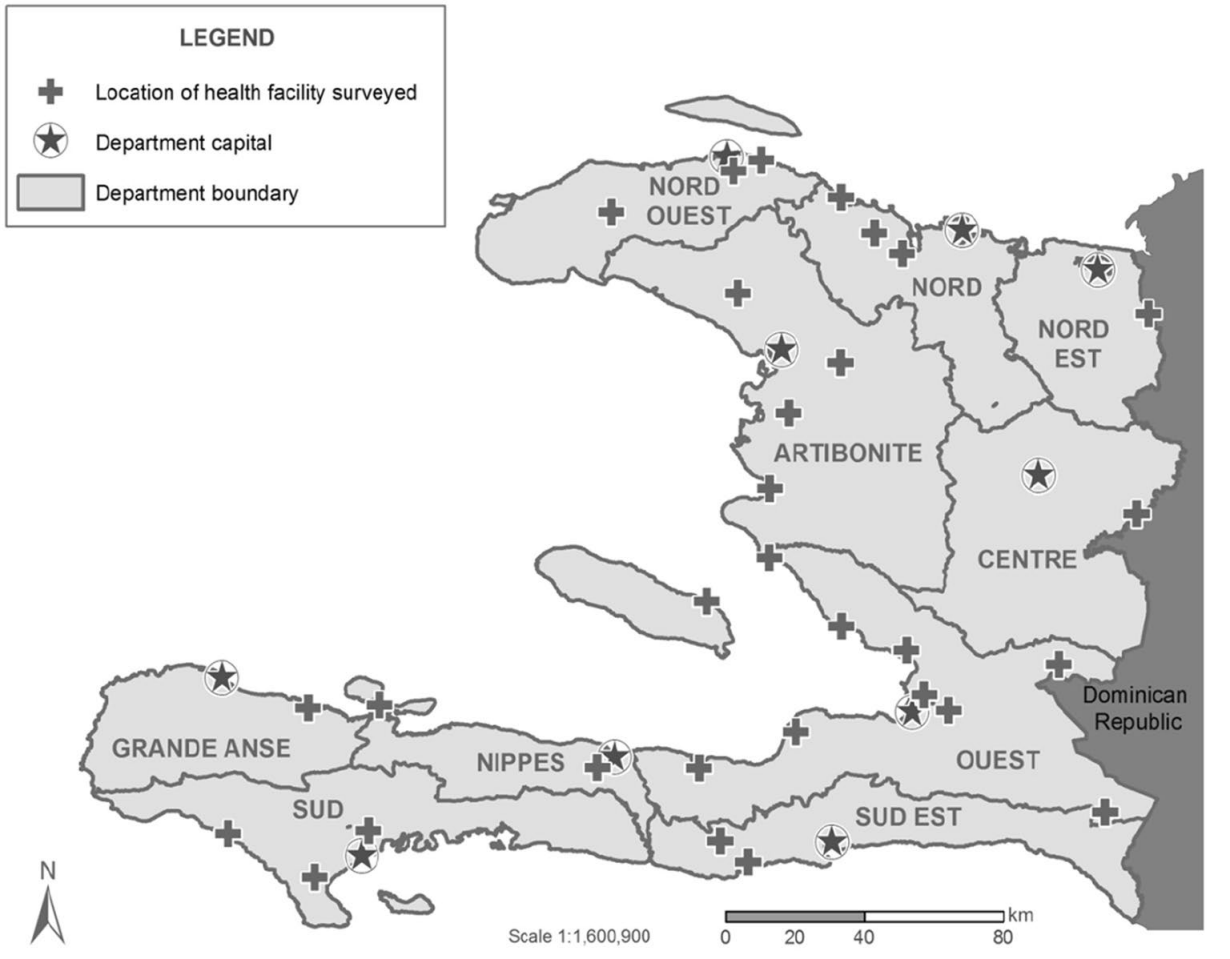
Map of sampled health facilities in Haiti

A sample size of 533 enrolled subjects was calculated to detect a malaria prevalence of 50 % with a precision of ±5 %, taking into account a type 1 error of 0.05, a design effect of 1.25, and a non-participation rate of 10 %. The malaria prevalence estimate was chosen to provide the most conservative estimate of sample size, and was informed by a post-earthquake survey which demonstrated a malaria prevalence of 20.3 % among febrile patients [6], much higher than had been observed in previous health facility surveys [3–5] which raised uncertainty about the true burden of malaria in this population. Thirty facilities were sampled across the 10 departments in Haiti (Fig. 2) to ensure an appropriate minimum number of units to account for clustering at the facility-level.

### Survey procedures

The field work for the survey was conducted between December 7 and 14, 2012. Each health facility visit was unannounced and took place over two consecutive days. On the first day, the survey teams conducted a quantitative inventory of the facility’s physical and human resources, and performed interviews with all available and consenting health care providers in the outpatient department. For the facility inventory, the survey teams utilized a standardized data collection instrument to assess resources in outpatient clinics, laboratories, and pharmacies. A second day was dedicated to an outpatient survey where patients presenting to the facility’s outpatient clinic were screened for fever. Eligible, consenting, febrile patients were enrolled and finger-prick blood samples were collected to prepare thick and thin blood smears from each subject. These blood smears were reserved for later interpretation at the LNSP, and were considered the gold standard. In addition, several drops of blood from each participant were collected onto Whatman 903 protein saver cards for PCR to assess sub-microscopic parasitaemia. Each patient was evaluated and treated per facility standard of care by the provider and laboratory. After the clinical encounter, the provider was asked to complete a short form on each study participant describing the diagnosis, tests ordered, and treatments prescribed for febrile illness, including anti-malarial drugs. Occasionally, if the provider was unable to fill out the form, it was completed by the survey team based on the clinical note completed by the provider. Enrolled patients were administered a post-consultation questionnaire to assess illness history, mosquito net ownership, and malaria knowledge.

### Laboratory procedures

Laboratory diagnosis of malaria was performed at two levels: (1) by the facility laboratory using microscopy if operational, or by RDTs if available; and (2) by the LNSP reference laboratory, which analysed survey-prepared blood smears from enrolled patients as the “gold standard”. Facility laboratory results were documented by the survey team if the laboratory completed and recorded the results by the end of the clinic day. Gold-standard microscopy was performed according to a standard protocol [13]. Briefly, after fixation of the thin smear in methanol, thin and thick smears were stained with 10 % Giemsa for 5–10 min. The survey-prepared blood slides, were double read by two expert, microscopists at the reference laboratory who were blinded to the facility laboratory results. The examination of slides began in January 2013, approximately 1 month after preparation in the field. In order to declare a microscopy specimen free of *Plasmodium falciparum* infection, 300–500 thick-smear fields were examined.

Dried blood spots, on Whatman 903 protein saver cards, were analysed in duplicate by LNSP in June 2014 by polymerase chain reaction using photo-induced electron transfer fluorogenic genus-specific primers (PET-PCR). Sample preparation, storage, extraction, and assays were performed using protocols described previously [10, 14], but modified to account for World Health Organization (WHO) Evidence Review Group recommendations made in March 2014 for PCR analysis in low transmission settings [15]. Briefly, the amplification of *Plasmodium* genus (5′–3′, forward primer: GCTCTTTCTTGATTTCTTGGATG; reverse primer: FAM-aggcgcatagcgcctgg AGCAGGTTAAGATCTCGTTCG) was performed in a 30 µL reaction containing 2X TaqMan Environmental buffer 2.0 (Applied BioSystems, Grand Island, NY, USA), 400 nm each of forward and reverse primers. For each sample, duplicate PET-PCR reactions were run with 6 µL of DNA template used in the PCR reaction with the following cycling parameters: initialization at 95 °C for 15 min, followed by 45 cycles of denaturation at 95 °C for 15 s, annealing at 63 °C for 60 s. The correct fluorescence channel was selected for FAM dye and the cycle threshold (CT) values recorded at the end of annealing step. All assays were performed using Applied Biosystems ABI 7500 or StepOne thermocyclers (Life Technologies, Carlsbad, CA, USA). Cycle threshold values, which are continuous, semi-quantitative measurements of parasite load, were obtained for all specimens. Those with CT values of less than 40.0 were considered positive for malaria.

Positive samples were subjected to additional testing by nested 18S rRNA PCR (nPCR) to confirm the *Plasmodium* species, utilizing a method previously described by Singh et al. [16]. Briefly, both primary and secondary PCR reactions were performed using 2 µL of DNA template in 25 µL total volume containing 1X buffer, 2.5 mM MgCl_2_, 200 µM dNTPs, 200 nM primers, and 1.25 units of Taq Polymerase (New England Biolabs, Ipswich, MA, USA). The products were analysed for appropriate band size on a 2 % agarose gel with a positive showing a single visible band.

### Data management and analysis

Data were entered using Epi Info™ 7 (7.1.1.14) (CDC, Atlanta, GA, USA) and Microsoft Excel for Windows 7 (Microsoft, Redmond, WA, USA). Analysis was performed with SAS 9.3 (SAS Institute, Cary, NC, USA) and involved simple counts and percentages. National estimates and 95 % confidence intervals (CI) were calculated using patient-level or health facility-level weights as appropriate using the SAS PROC SURVEYFREQ procedure in order to account for the complex sampling design. Sensitivity and specificity analyses were conducted comparing facility diagnostic results to the survey-performed gold-standard microscopy. The map was created using ARCGIS 10.2.2 (Esri, Redlands, CA, USA) using spatial data of health facilities courtesy of the Haitian Institute of Statistics and Informatics (Institut Haïtien de Statistiques et d’Informatiques) and National Center of Geo-spatial Information Systems (Centre National de l’Information Géo-Spatiale).

### Definitions

For the purposes of this survey, health facilities were defined as having the capacity to diagnose malaria if they fulfilled the following criteria: (1) a facility laboratory completed a malaria test on the survey day for at least one enrolled patient using either an RDT or a microscopy examination of a blood smear, or (2) the survey inventory results determined that there was on-site capacity to perform either microscopy (functional microscope, reliable electricity, staining reagents, and a trained laboratory technician) or RDTs (current stock of any one of the three MSPP-approved RDT brands and at least one employee trained in performing RDTs). Reliable electricity was defined as having electricity for four or more hours a day, 5 days a week or having at least one generator in the hospital.

“Treatment according to guidelines” referred to directives set in the MSPP 2012 malaria treatment policy and was defined as: (1) prescription of the correct dose of chloroquine and primaquine to those with a positive malaria test result, and (2) no prescription of anti-malarials to those with a negative test result [1]. For treatment doses, the total adult regimen is 1500 mg of chloroquine base (administered over 3 days, with four tablets containing 150 mg each given on day one, and three tablets given on days two and three) and a single dose of 0.75 mg/kg primaquine (maximum adult dosage is 45 mg) on the first day of treatment. Paediatric regimens are adjusted according to age and weight [1]. The MSPP/PNCM supplies tablets containing 150 mg of chloroquine base and 7.5 mg tablets of primaquine.

### Human subjects review

The survey protocol and questionnaire were reviewed and approved by the human subjects review boards of the MSPP (reference number 1112-30) and the US Centers for Disease Control and Prevention (Atlanta, GA, USA). Written consent was obtained from each subject, or from guardians accompanying subjects younger than 18 years; children 7–17 years old provided written assent to participate.

## Results

### Facility and patient characteristics

**Fig. 3:**
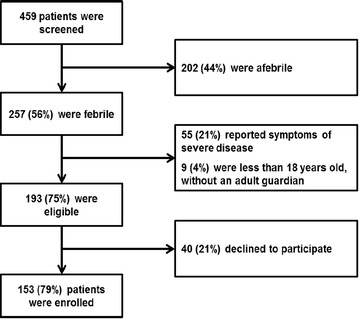
Flow chart of patient enrolment

Figure 2 shows a map of the thirty health facilities sampled proportionally across all ten Departments; of these 19 offered outpatient services only (14 were *dispensaires*, and five were *centres sans lits*); 11 facilities offered both outpatient and inpatient services (five were *centres avec lits,* and six were *hôpitaux*). Figure 3 shows the flow chart for participant enrolment. Four hundred and fifty-nine patients presenting to the sampled outpatient clinics on the day of the survey were screened for inclusion. Two hundred and fifty-seven (56 %) patients had either an axillary temperature ≥37.5 °C at evaluation or a history of fever. Of these, 64 were ineligible because they were below the age of consent and did not have a guardian present (n = 9, 4 %) or because they had symptoms consistent with severe illness (n = 55, 21 %). Of the 193 eligible patients, 40 (16 %) patients refused, resulting in 153 (79 %) enrolled patients.

Table 1 describes the participant characteristics. The mean age of enrolled patients was 22.6 years, median was 22 years, with a range of 3 months to 80 years old. It was more common for patients to be female (n = 103, 68 %), and 13 women were pregnant (9 % of all participants). Sixty-one percent of patients owned any mosquito net (n = 94), and of these, 63 reported sleeping under a net the previous night (68 %). More than two-thirds of patients (or caregivers) correctly indicated that mosquitoes transmit malaria (n = 104, 68 %). Additional patient characteristics are shown in Table 1.

**Table 1:** Enrolled patient characteristics

Characteristic	n/N (%)
Aged <5 years	39/146 (27)
Female	103/152 (68)
Pregnant	13/149 (9)
Live in urban area	64/148 (43)
Days symptomatic	
≤3 days	47/151 (31)
4–7 days	51/151 (34)
8–14 days	30/151 (20)
>14 days	23/151 (15)
Days febrile	
≤3 days	53/146 (36)
4–7 days	49/146 (34)
8–14 days	28/146 (19)
>14 days	16/146 (11)
Own a mosquito net	94/153 (61)
Slept under mosquito net last night	63/92 (68)
Knows that mosquitoes transmit malaria	104/153 (68)
Sought care at health facility first	100/152 (66)
Education	
Primary school or less	84/152 (55)
Secondary school or more	68/152 (45)
Time to travel to facility	
<30 min	71/151 (47)
30–60 min	36/151 (24)
1–2 h	38/151 (25)
>2 h	6/151 (4)
Satisfied with care received	128/149 (86)

*N* number of responses

### Provider characteristics

One hundred and fifteen providers were interviewed, of whom 102 provided direct patient care and were included in our analysis. Providers reported a mean 4.3 years of medical training (range 0–12, median of 3), and 61 (60 %) providers were female. Fifty (49 %) providers had received in-service training in the case management of malaria or severe malaria, and 16 (14 %) had this training in 2012, after the malaria treatment guidelines had been revised.

### Malaria case management

Malaria diagnostic capacity of health facilities is shown in Table 2. Eleven (37 %) facilities surveyed had diagnostic capacity on the basis of having completed malaria testing of at least one patient. According to inventory results, eight (27 %) and two (7 %) facilities had the materials and personnel necessary to conduct either microscopy or RDT, respectively. Eighteen (60 %) facilities reported having electricity at least 4 h a day, 5 days a week; six (20 %) facilities had neither electricity nor a generator. Adequate supplies and equipment for microscopy were present in 11 (37 %) facilities. Three (10 %) facilities had at least one brand of RDT in stock (≥1 test) on the day of the survey. Of these, two had an approved brand and two had non-approved brands; one facility had both an approved and a non-approved brand in stock. Five (19 %) facilities previously had RDTs available, but were out of stock on the day of the survey. Among these facilities, three (60 %) used an approved brand, one used a non-approved brand, and one could not specify the brand. Thirteen facilities (43 %) had at least one provider or laboratory technician trained to perform RDTs. Altogether, 16 (53 %) of 30 facilities had the ability to diagnose malaria, and 91 (59 %) enrolled patients were seen at facilities with this capacity.

**Table 2:** Facilities with malaria diagnostic capacity

Definition	Criteria for malaria diagnostic capacity	Facilities n (%)
1	Test conducted during the survey: ≥1 blood smear or RDT done and results available in facility laboratory	11 (37)
2	Malaria microscopy capacity: adequate laboratory and electrical supply	8 (27)
	Adequate laboratory supplies and equipment (≥1 working microscope, Giemsa stain and glass slides)	11 (37)
	Adequate electrical supply (electricity at least 5 days/week and ≥4 h/day or generator)	18 (60)
	Trained microscopy technician	16 (53)
3	RDT diagnosis: stock of an approved RDT and >1 health worker trained on them	2 (7)
	≥1 approved RDT in stock (CareStart, First Response, Bioline)	2 (7)
	≥1 provider trained in performing RDTs	13 (43)
1, 2, 3	Diagnostic capacity by any definition	16 (53)

Training on malaria microscopy, RDTs and current MSPP malaria guidelines was reported by the facilities, according to health worker cadre (health care provider or laboratory technician). Trained microscopy laboratory technicians were staffed at 16 (53 %) facilities (Table 2). For RDTs, a total of 13 (43 %) facilities had at least one staff (of any cadre) trained; seven facilities had at least one laboratory technician trained, nine facilities had at least one clinical health provider trained, and three facilities had both laboratory technicians and health providers trained on RDTs. Fifteen facilities (50 %) reported that personnel (of any cadre) were trained on the malaria treatment guidelines. Of these, 14 facilities had at least one clinical health provider trained; seven facilities had both providers and laboratory technicians trained on the new guidelines, and one facility had only a laboratory technician trained on the new guidelines.

**Fig. 4:**
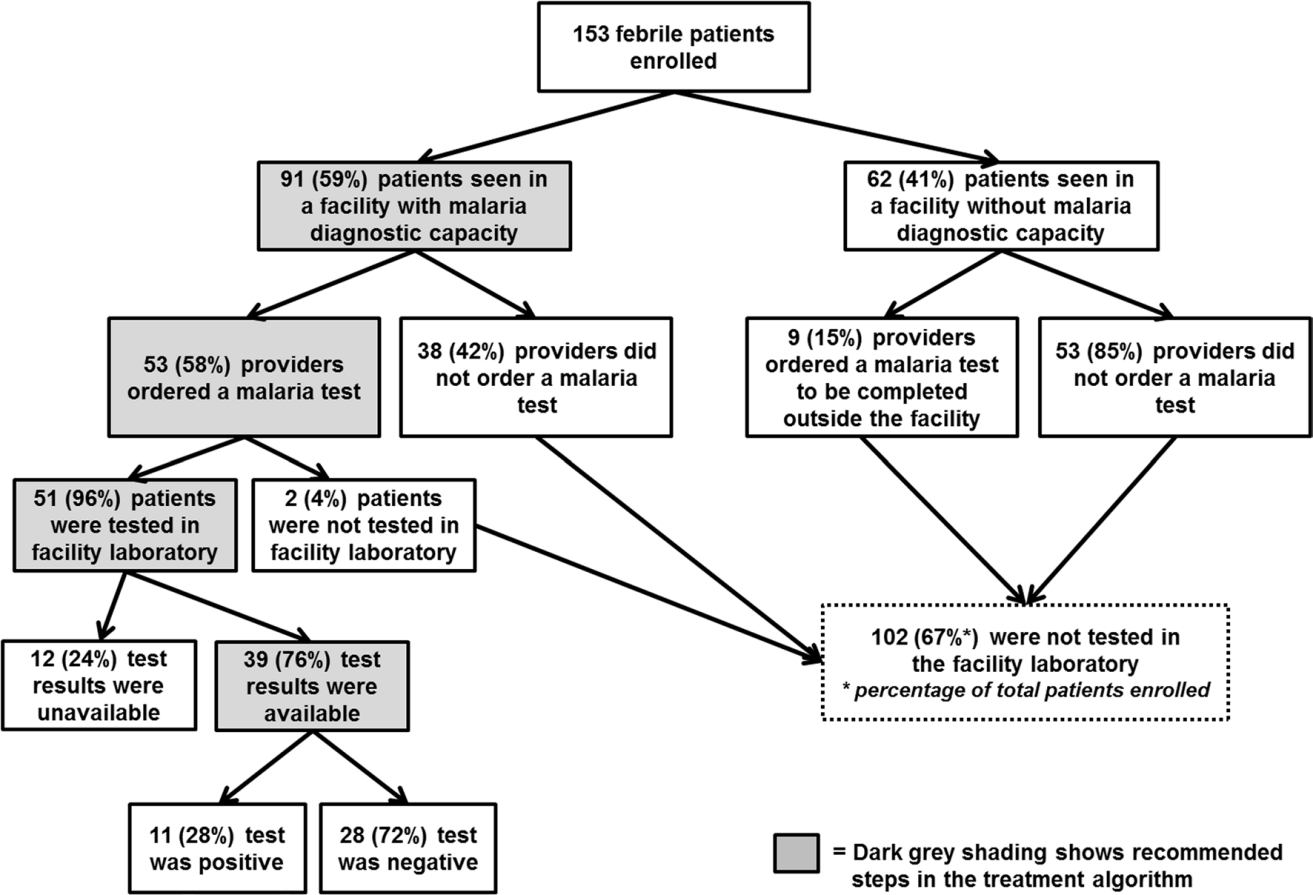
Flow chart for malaria testing

Data on the ordering and completion of malaria tests are shown in Fig. 4. Malaria test ordering practices were assessed using the post-consultation form completed by health care providers. Among the 91 febrile patients seen in 16 facilities with malaria diagnostic capacity, 53 (58 %) had a malaria test ordered by a provider (45 [50 %] microscopy, and 16 [18 %] RDT). At 14 facilities without diagnostic capacity, nine (15 %) of 62 patients had an order written for a test to be done at an outside laboratory: all nine included an order for microscopy, and eight included an RDT order.

Laboratory registers at facilities with diagnostic capacity were reviewed to obtain malaria test results for enrolled patients (Fig. 4). Fifty-one (96 %) of the 53 patients with an order for a diagnostic test had a test initiated in the facility laboratory, and 39 (76 %) of these patients had a laboratory result recorded at the end of the survey day, in time for clinical decision-making. Eleven (28 %) patients with completed tests were found positive by the facility for malaria. Malaria diagnostic tests were not done for 102 (67 %) of the enrolled patients.

Among the 51 patients tested for malaria, 56 tests were initiated (five patients had both a microscopy and RDT test performed), including 46 smears and ten RDTs. Of the smears, 24 (52 %) were read as negative by the facility laboratory, ten (22 %) were read as positive, and 12 (26 %) did not have results recorded in the laboratory results register by the end of the survey day and were considered unavailable for clinical decision-making. Of the ten RDTs performed, nine (90 %) were negative and one (10 %) was positive. The patient with a positive RDT did not have a blood smear performed in the facility laboratory, and the brand of the RDT used was not one of the approved brands.

The 2012 malaria treatment guidelines made two fundamental changes to the treatment recommendations: (1) only confirmed patients should be treated with an anti-malarial; and (2) a single dose of primaquine is given, in addition to the 3-day regimen of chloroquine.

**Fig. 5:**
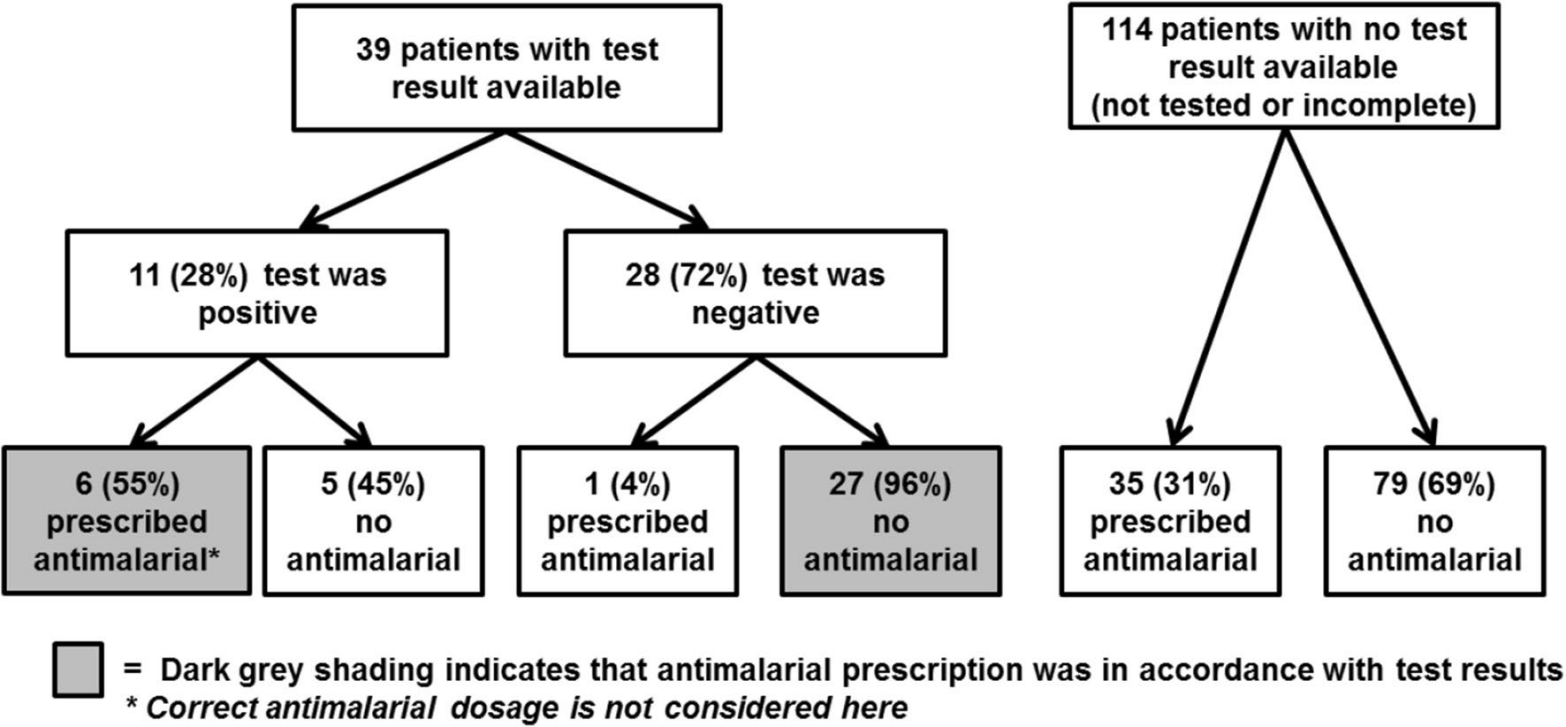
Flow chart of testing results and antimalarial treatments

To assess treatment decisions, anti-malarial prescriptions are shown in Fig. 5 for two groups: patients with test results available in time for clinical decision-making (n = 39), and patients without a test result (n = 114), either because a test was initiated but results were unavailable (n = 12) or because no test was done (n = 102). Among 39 patients with results available, 33 (85 %) were treated in accordance with their test result. However, five (45 %) of 11 patients with a positive test result from the facility laboratory were not treated with an anti-malarial, and one (4 %) of 28 patients with a negative test was treated with an anti-malarial. Of the 114 patients with no test results, 35 (31 %) were treated presumptively with an anti-malarial.

A total of 42 (27 %) patients were prescribed any anti-malarial, and all of them included chloroquine. Six (4 %) patients were also prescribed primaquine, of which one (17 %) was prescribed the correct adult dosage (45 mg), and three were prescribed a higher dose according to age and weight charts. In all cases of incorrect prescription of primaquine, the tablet size documented (75 mg as primaquine phosphate salt) was similar to the recommended total milligrams-per-kilogram dose of primaquine (0.75 mg/kg). Since primaquine is not available in tablets of this size, it is unclear if these reported overdoses represent true overdoses or reporting error. Of all patients treated with any anti-malarial, only one (2 %) was treated with a correct dose of chloroquine and primaquine according to the national guidelines.

### Gold-standard microscopy results, PCR, and sensitivity and specificity of facility testing

The reference laboratory examined 153 gold-standard blood smears prepared by the survey team, of which 13 could not be read due to poor staining or fixation, or due to degradation of the stain in the interval period. Of the remaining 140, none were positive for any *Plasmodium* species. Specimens from 33 patients had results available from both the gold-standard survey microscopy and the facility laboratory. However, since no ‘true positives’ were identified by the survey microscopy, it was not possible to calculate the sensitivity for facility-read blood smears. Specificity of the facility results was 70 % (95 % CI 51–84 %).

PET-PCR was performed at LNSP on dried blood spots from each of the 153 enrolled patients. One sample was positive with duplicate CT values of 39.0 and 39.1, which are just under the CT cutoff threshold for positivity of ≤40. Calibration assessments using the PET-PCR protocol described herein have determined that a CT value of 34 corresponds to approximately 100 parasites per microlitre, and therefore specimens with CT values between 34 and 40 are generally considered below the threshold of detection by traditional microscopy and most commercial RDTs (unpublished observations, E. Rogier, JW Barnwell, V. Udhayakumar). Nested PCR was conducted at the CDC-Atlanta malaria laboratory and confirmed that this specimen contained a single-species infection with *P.falciparum*. The patient from whom the sample had been obtained was a 28-year old male seen in a facility in Port-au-Prince, who had previously sought care elsewhere and may have had prior treatment. On the survey day, a blood smear and RDT were ordered; the RDT was positive, but the facility microscopy result was not available. The patient was treated with chloroquine alone on the survey day. The gold standard blood smear was negative.

### National estimates

Table 3 shows the national estimates calculated for key indicators. The national estimate of the prevalence of malaria among patients seeking care at health facilities, detected by PCR, was 0.5 % (95 % CI 0.0–1.7). Two criteria were used to classify patients as being managed by the health facilities according to 2012 national guidelines: (1) those with positive malaria test results (from the facility) and prescribed the correct dose of chloroquine and primaquine (n = 1), and (2) those with negative test results not prescribed any anti-malarial (n = 27). The national estimate for febrile patients being treated according to the 2012 guidelines is 16 % (95 % CI 0.0–39). Patients with malaria results available for clinical decision-making was estimated at 17 % (95 % CI 0.0–40).

**Table 3:** National estimates of key indicators

Indicator	Point estimate (%)	95 % confidence interval
Patients with malaria detectable by PCR	0.5	0–1.7
Patients managed according to 2012 national guidelines	16	0–39
Patients with malaria test result available for clinical decision-making	17	0–40
Facilities with malaria diagnostic capacity	56	36–77
Facilities having a provider trained in RDT use	45	26–65
Health providers trained on RDTs	23	11–36
Health providers trained in 2012 on malaria treatment guidelines	16	9–23

Health facilities with diagnostic capacity for malaria were estimated at 56 % (95 % CI 36–77), and 45 % (95 % CI 26–65) of health facilities were estimated to have a provider trained to use RDTs.

Twenty-three percent (95 % CI 11–36) of health providers were trained on RDTs, and 16 % (95 % CI 9–23) of them were trained on malaria treatment guidelines in 2012.

## Discussion

This study provides baseline findings on the quality of malaria case management in Haiti and the health system factors affecting it, prior to implementation of national training and distribution of malaria treatment guidelines and RDTs. Critically, a low proportion (53 %) of facilities had diagnostic capacity for malaria (Table 2). While the possession of microscopes among health facilities was relatively high (70 % data not shown), unreliable electricity and an inconsistent supply of reagents and slides contributed to the low capacity of facilities to diagnose malaria by microscopy. Similarly, only two (7 %) facilities had stock of an approved RDTs for malaria diagnosis.

Additionally, there was a substantial volume of presumptive malaria treatment without laboratory-confirmed diagnosis—almost one third of patients not tested for malaria were treated with an anti-malarial (Fig. 5). However, as malaria is responsible for a minority of fevers in Haiti it is necessary to confirm cases of malaria with high-quality diagnostic tests prior to providing treatment. The utilization of RDTs, which can be performed in the absence of a laboratory and read at the point of care, may reduce these missed opportunities for malaria confirmation, but access to this commodity needs to be improved. Presumptive malaria treatment impedes diagnosis and treatment of the true cause of febrile illness, and results in an overestimate of malaria burden, which affects prioritization of resources for public health interventions.

Conversely, 45 % of patients in this survey who tested positive for malaria at the facilities were not treated with an anti-malarial on the survey-day (Fig. 5). It is possible that inefficient intra-facility communication between the laboratories and providers may have contributed to the discrepancy between the positive malaria test results and the lack of anti-malarial prescriptions. Continual training on the updated malaria treatment guidelines should emphasize to providers the importance of using the results from malaria tests, especially RDTs, on the first day of the clinical visit to inform diagnoses and medication prescriptions. Facility and regional leadership should be engaged to find local solutions to intra-facility communication problems, and promote all clinical staff to adhere to the malaria guidelines to test and treat on the day of clinical presentation.

In this survey, facility laboratory results showed a high number of false positive tests: none of the 11 patients who tested positive (10 according to microscopy tests, and 1 according to RDT) for malaria at the facility-level had smear-confirmed malaria according to the national reference laboratory. Earlier health facility-based surveys demonstrated low positive predictive values of Haitian health facility microscopy for malaria ranging from 22 to 40 % [4, 5], thus the current findings suggest that the lack of proficient microscopy in many health facilities in Haiti is an ongoing problem. Given the resources necessary to develop and maintain expertise to conduct widespread microscopy for malaria diagnosis, RDTs may be the best choice to extend malaria diagnostic capacity in Haiti, as in other low-transmission settings [17].

Notably, the one RDT positive result was confirmed by PET-PCR as a low-density malaria infection, resulting in a national estimate of malaria prevalence among febrile outpatients of 0.5 (95 % CI 0–1.7 %). This sample was negative by microscopy. There are several possible explanations for this discrepancy. First, multiple studies have demonstrated that PCR is far more sensitive than microscopy, especially in contexts of lower levels of malaria transmission; in one meta-analysis, among studies where population malaria prevalence was <10 %, microscopy detected only 12 % of infections detected by PCR [18]. However, given the processing time, cost, and demand for specialized equipment, trained personnel, and steady electrical supply, PCR is not practical as a diagnostic tool, especially in low resource settings. Second, RDTs detect circulating parasite antigen which can remain after an active infection is cleared, and thus it is possible for RDTs to detect a recent infection in an individual clinically cured within the previous 2 weeks; this limitation could be partially resolved if providers ask about recent treatment for malaria. There is a suggestion that due to this mechanism, RDTs are more likely than microscopy to detect low-density infections [19].

All anti-malarial regimens prescribed by health workers in this survey included chloroquine. However, a minority of patients were also treated with single-dose primaquine, which was recently added to the first-line regimen in the 2012 national guidelines. Overall, only one of 42 patients prescribed an anti-malarial was treated with the correctly dosed regimen of chloroquine plus primaquine.

According to study records, three of the six patients prescribed primaquine had documented doses greater than those recommended for age and weight, likely attributable to confusion between tablet size and total recommended dosage. Ongoing provider training should clarify the primaquine dosing and tablet sizes available to avoid potential overdoses, which can be especially dangerous to individuals with glucose-6-phosphate dehydrogenase deficiencies.

This evaluation has several limitations. In accordance with the design of this study, only persons who presented to health facilities on the survey day were evaluated. Access to health care is a pervasive problem in Haiti, with 82 % of women aged 15–49 years claiming at least one barrier to accessing care (permission to get care, money for treatment, distance to a facility, not wanting to go alone) [20]. In addition, available data did not permit selection of health facilities on the basis of utilization, although this limitation was minimized in the calculation of national estimates by weighting the final sample according to utilization rates ascertained during survey procedures.

It is possible that some portion of the over-diagnosis and over-treatment observed could be attributable to the Hawthorne effect related to health care workers’ knowledge of the presence of surveyors associated with the malaria programme [21]; although the survey was not announced prior to the arrival of the survey teams, health care workers were aware of the teams’ affiliation and may have wanted to appear vigilant with respect to the evaluation and treatment of malaria. However, this phenomenon would not have impacted the gold standard blood smear results.

An additional limitation is the relatively large number of screened patients who were excluded due to self-report of symptoms of severe disease. Despite efforts to correctly translate severe symptoms into Haitian Creole, there may have been misinterpretation of the translated medical terms; omission of these patients may have resulted in an underestimation of malaria cases in general, and severe malaria in particular. Exclusion of patients for other reasons, including refusal to participate, may have resulted in participation bias. Finally, although effort was made to minimize differences in survey teams’ methods, missing data, reporting inaccuracies, and variability of methods between teams may also have affected this study’s internal validity.

## Conclusions

This survey found high levels of clinical diagnosis and presumptive treatment of malaria by health workers, as well as low levels of correct anti-malarial prescription for those patients diagnosed with malaria. The reasons for these findings likely include poor diagnostic capacity and a low level of health care provider training for malaria diagnostics and treatment on the revised guidelines. Priorities for the malaria programme and partners going forward include wide-reaching training on and implementation of a limited number of high-quality, easy-to-use RDT brands, and improvement in provider training and supervision on Haiti’s updated treatment guidelines. This study provided a baseline estimation of health system factors contributing to malaria case management in Haiti, prior to programme scale-up, and highlights opportunities to direct programme resources for improved performance. A subsequent health facility survey was conducted in late 2014 to assess programme progress.

## Abbreviations

CDC: US Centers for Disease Control and Prevention
CQ: chloroquine
CT: cycle threshold
GFATM: global fund to fight AIDS, tuberculosis and malaria
LNSP: Laboratoire National de Santé Publique, (National Public Health Laboratory), or reference laboratory
RDT: rapid diagnostic test
MSPP MSPP: Ministère de la Santé Publique et de la Population, (Ministry of Public Health and Population)
PCR: polymerase chain reaction
PET-PCR: PCR using photo-induced electron transfer fluorogenic primers
nPCR: nested primer PCR
PNCM PNCM: Programme National de Contrôle de la Malaria (National Malaria Control Programme)
PSI/OHMaSS: Population Services International/Organisation Haïtienne de Marketing Social pour la Santé
PQ: primaquine
